# Determinants of birth asphyxia among newborns in south Gondar Zone public hospitals, North West Ethiopia, 2021: A case control study

**DOI:** 10.1016/j.heliyon.2024.e30093

**Published:** 2024-04-25

**Authors:** Eyob Shitie Lake, Zinie Abita, Besfat Berihun Erega

**Affiliations:** a Department of Midwifery, College of Medicine and Health Sciences, Woldia University, Woldia, Ethiopia; b School of Public Health, Mizan Tepi University, Mizan Teferi, Ethiopia; c Department of Clinical Midwifery, Collage of Medicine and Health Science, Debre Tabor University, Debre Tabor, Ethiopia

**Keywords:** Birth asphyxia, Determinants, South Gondar zone, Case control

## Abstract

**Introduction:**

Birth asphyxia is one of the leading causes of neonatal mortality, which accounts for around 24 % of overall neonatal mortality. Neonatal death usually results from preventable factors. Thus, this study has aimed to identify the determinant factors of birth asphyxia among newborns in South Gondar Zone public hospitals.

**Methods:**

Institution based unmatched case control study and systematic random sampling technique was conducted in South Gondar zone public hospitals from March October 2021 to May 20/2021. A pretested interviewer administered questionnaire and a data retrieving checklist was used for data collection. Cases were selected if one of the following was present at birth: (gasping, no breathing, or breathing rate of below 30 per minute). Epidata version 4.6 software was used for data entry and bivariate logistic regression and multivariable logistic regression techniques were used for data analysis using SPSS version 23.

**Result:**

In this study, Instrumental delivery (AOR = 3.19, 95%CI: 1.23–8.36), labor abnormality (AOR = 3.24, 95%CI: 1.31–8.03), cord prolapse (AOR = 7.06, 95%CI:2.25–22.50),APH (AOR = 4.68,95%CI:2.00–10.95) and preterm birth (AOR = 3.84,95%CI:1.32–11.20) were predictors of birth asphyxia.

**Conclusion:**

Labor abnormality, ante-partum hemorrhage, cord prolapse, instrumental delivery and preterm birth were independent predictors of birth asphyxia.

## 1 Introduction

Birth asphyxia is described as the inability to commence, establish, and maintain breathing during childbirth due to a lack of oxygen, persisting for a significant duration in the birthing process for a newborn infant [1]. Despite a decrease in worldwide neonatal mortality rates, around three million infants still succumb to death each year within their initial 28 days of life, primarily occurring in the first day and within the first week. Notably, perinatal asphyxia contributes to 24 % of the causes of neonatal deaths [2].

The global neonatal mortality rate has persistently decreased, following a prolonged decline from 31 deaths per 1000 live births in 2000 to 18 per 1000 in 2017. Approximately 7000 neonatal deaths occur daily, with one-third of them happening on the first day of life [3]. In Brazil, early neonatal deaths linked to birth asphyxia constituted 40 % of all early neonatal deaths among infants weighing ≥2500 g without malformations [4]. Nevertheless, the weight of neonatal mortality in sub-Saharan Africa persisted as indicated by a neonatal mortality rate of 27.2 per 1000 live births. A child born in sub-Saharan Africa or Southern Asia faces a tenfold higher likelihood of death within the first month compared to a child born in a high-income country [3].

Of those admitted to neonatal intensive care unit (NICU), the majority of neonatal mortality were caused by birth asphyxia followed by severe neonatal infections [5]. In Ethiopia, perinatal asphyxia stands out as one of the most prevalent reasons for neonatal admissions to the Neonatal Intensive Care Unit (NICU), and a high case fatality rate has been observed among neonates diagnosed with perinatal asphyxia [[5], [6], [7]]. The neonatal mortality pattern among preterm infants admitted to the University of Gondar Comprehensive Specialized Hospital revealed that 28.8 % of neonates did not survive. Among them, 11.4 % succumbed within the initial 24 h of life. Consequently, birth asphyxia accounted for more than one-third of these neonatal deaths [8].

Even though the coverage of antenatal care and skilled delivery has been increasing in Ethiopia, the neonatal mortality rate remained unchanged; this is 29 and 30 per 1000 live births in 2016 and 2019 respectively. Furthermore, the problem worsens in Amhara region, 47 per 1000 live births, which is far from the sustainable development goal target of reducing neonatal mortality rate to 12 or fewer per 1000 live births by 2030[9].

The tragedy of birth asphyxia is not only limited to neonatal deaths, but also associated with short term and long-term health consequences. For instance, multi-organ failure is common in infants born after acute or prolonged asphyxia [10]. In a retrospective study of children aged two years and above conducted using the Canadian Cerebral Palsy Registry, findings indicated that perinatal asphyxia exacerbates cerebral palsy. Specifically, 41 % of individuals with cerebral palsy following neonatal encephalopathy had a history of perinatal asphyxia. Furthermore, these cases accounted for 58 % of non-ambulatory children and 55 % of children diagnosed with spastic quadriplegia [11]. Children who have experienced birth asphyxia often exhibit acute brain injury, leading to cognitive, sensory, and motor impairments. This is further reflected in their academic performance, with many struggling and requiring special education support to successfully graduate from school [12,13].

Neonatal mortality rate in Ethiopia remained unchanged after 2016, which is 29 and 30 per 1000 live births in 2016 and 2019 respectively. Furthermore, the problem worsens In Amhara region, 47 per 1000 live births, which is far from the sustainable development goal target of reducing neonatal mortality rate to 12 or fewer per 1000 live births by 2030. Studies about determinant factors of birth asphyxia in our study setting are limited, since there is socio-demographic difference. Variables such as WHO safe childbirth checklist and labor abnormalities previous were not studied in our country. Our study had filled this gap by incorporating these variables as part of the study.

As per our review, previous studies had used neonates admitted to neonatal intensive care unit with a diagnosis of perinatal asphyxia as cases and non-asphyxiated but admitted with sepsis, jaundice, hemorrhage and congenital anomalies as controls. So, unlike this study data was collected from newborn babies diagnosed as having birth asphyxia as cases and non-CCCasphyxiated newborns as controls in the labor ward, helps not to miss those asphyxiated neonates who responded for resuscitation in labor ward but not admitted at NICU. Thus, our study has aimed at identifying determinant factors of birth asphyxia among newborns in South Gondar Zone public hospitals.

## 2 Methods

### 2.1 Study design and period

Institution based unmatched case control study was conducted from March 10/2021to May 20/2021 in South Gondar Zone, North West Ethiopia. The study was conducted at Debre Tabor referral hospital, Nefas-Mewcha primary hospital, Addis Zemen primary hospital and Mekane-yesus primary hospital.

### 2.2 Study participants

All live newborns after 28 completed weeks with their respective mothers in South Gondar zone public hospitals were the source population and all live newborns after 28 completed weeks with their respective mothers in selected hospitals of south Gondar zone during the data collection period. In this study, neonates were categorized in to cases and controls.

### 2.3 Eligibility criteria

#### 2.3.1 Inclusion criteria

Cases: Neonates who had one of the following signs (gasping, not breathing, breathing rate below 30 per minute) at birth were defined as having birth asphyxia.

Controls: Neonates who had no signs of breathing difficulty were recruited as controls.

#### 2.3.2 Exclusion criteria

• Newborns who lost their mothers during or immediately after birth
• Newborns whose mothers were critically ill and cannot communicate well.
• Newborns with one or more major congenital malformations and incompatibility with life, such as hydrops and cyanotic congenital heart defects

#### 2.3.3 Sample size determination and sampling technique

The sample size was calculated by Epi info 7 software for the unmatched case control study. The largest sample size was calculated by the assumption of 95 % confidence level, a power of 80 % and the proportion of asphyxia among controls born after prolonged labor (a key predictor of birth asphyxia) was 34.7 % with an odds ratio of 2.00(9), the final sample size was 329 with a case to control ratio of 1:2 (110 cases and 219 controls). Based on the previous four months delivery flow rate; an average of 1455 deliveries and 328 asphyxiated newborns are expected during the data collection period. After proportional allocation to each hospital was done, every third asphyxiated neonate was selected as a case, while every 5th non-asphyxiated newborn baby was enrolled as a control. It was taken from the delivery registration book and a lottery method was employed to select the first study participant for case as well as controls. Thus, the first second asphyxiated baby and the third non asphyxiated were randomly selected and first recruited for the study.

#### 2.3.4 Data collection tools and procedures

A pretested interviewer administered structured questionnaire and data retrieving checklist were used to collect data after pretested with 5 % at Felege-Hiwot specialized referral hospital. The questionnaire was adapted from previous studies [13,14]. Data was collected by a total of four BSc midwives and a total of four supervisors were supervising the data collection procedure. The Amharic version of the questionnaire was used to collect the socio-demographic characteristics and antenatal characteristics, while the English version data retrieving checklist was used to collect the intra-partum and neonatal factors.

#### 2.3.5 Operational definitions

● Adverse birth outcome: one or more of the following: miscarriage(spontaneous termination of pregnancy before 28 weeks of gestation), stillbirth (gave birth with no sign of life), low birth weight (a baby born with a birth weight of below 2500 g), early neonatal death (death of neonate with in the first seven days of life) or preterm birth (birth of a baby before 37 weeks of gestation) [15].
● Birth asphyxia: if the newborn has one of the following signs: Not breathing, gasping, breathing poorly (<30 breaths/minute) [16].
● Labor abnormality: the presence of either or more of the following: prolonged latent first stage of labor, protracted cervical dilation, arrest of cervical dilation or prolonged second stage of labor.
● Nutritional status of the mother: mothers with mid-upper arm circumference (MUAC) of below 22 cm are undernourished and mothers with MUAC of 22 cm and above are normal [17].
● Maternal pyrexia: a maternal temperature of more than 99.9^0^ F during labor and delivery [18].
● Safe childbirth checklist utilization: at least the first three components (at admission, immediately before second stage of labor and immediately after birth) of the checklist were completed and used by reviewing the chart after birth [19].

#### 2.3.6 Data quality assurance and analysis

Data collection was started after the questionnaire was pretested by 5 % of sample size and necessary modification was done accordingly. One day training was given for the data collectors and supervisors on data collection process. Data collection process was strictly followed day to day by the supervisors and principal investigator and the collected data were checked for completeness and consistency every day by supervisors. Before analysis, data were cleaned up and cross checked and missing values were coded. The data were entered in to epi data version 4.6 and then exported to SPSS version 23 for analysis. Both descriptive and analytical statistical procedures were utilized. Binary logistic regression was used to identify factors associated with outcome variables. Multivariable logistic regression model was fitted to control the possible effect of con founders. Variables having *P*-value less than 0.2 in the bivariate analysis were fitted to multi-variable logistic regression models. Then the adjusted odds ratio (AOR), its 95 % CI interval and p-value ≤0.05 were considered to determine the statistically significant association.

## 3 Result

### 3.1 Socio-demographic characteristics of the respondents

A total of 329 neonates to maternal pair (110 cases and 219 controls) participants were involved in our study with a response rate of 100 %. The mean age of the cases and controls of participants were 27.74 (SD = 6.456) and 26.92 (SD = 6.438) years respectively. More than half of the mothers (65.5 %) of asphyxiated neonates and 59.8 % of mothers of non-asphyxiated neonates were from urban area. Among all the study participants, 94.5 % of cases and 93.2 % of controls were married (Table 1).

**Table 1:** Socio-demographic characteristics of the respondents among newborns in south Gondar zone public hospitals, Northwest Ethiopia, 2021 (n = 329).

Variables	Cases(%) (n = 110)	Controls(%) (n = 219)	Total (n = 329)
Age in years			
<20	12(10.9)	23(10.5)	35(10.6)
20-34	81(73.6)	164(74.9)	245(74.5)
35-49	17(15.5)	32(14.6)	49(14.9)
Place of Residence			
Urban	72(65.5)	131(59.8)	203(61.7)
Rural	38(34.5)	88(40.2)	126(38.3)
Marital status			
Single/divorced/widowed	6(5.5)	15 (6.8)	21(6.4)
Married	104(94.5)	204(93.2)	308(93.6)
Educational level			
Unable to read and write	35(31.8)	52(23.7)	87(26.4)
Able to read and write	17(15.5)	53(24.2)	70(21.3)
Primary school	23(20.9)	51(23.3)	74(22.5)
Secondary school	17(15.5)	28(12.8)	45(13.7)
College and above	18(16.4)	35(16.0)	53(16.1)
Occupation			
Housewife	46(41.8)	92(42.0)	138(41.9)
Farmer	28(25.5)	76(34.7)	104(31.6)
Merchant	17(15.5)	21(9.6)	38(11.6)
Government employee	15(13.6)	24(11.0)	39(11.9)
Others	4(3.6)	6(2.7)	10(3)
Average Monthly income in ETB			
≤2000	44(40)	105(47.9)	149(45.3)
2001-5000	32(29.1)	63(28.8)	95(28.9)
>5000	34(30.9)	51(23.3)	85(25.8)

Others = students and daily laborers, ETB = Ethiopian Birr.

### 3.2 Ante-partum related characteristics of the respondents

The findings of this study showed that thirty-three (30 %) mothers of asphyxiated neonates and around one-third (34.2 %) mothers of non-asphyxiated neonates had experienced the pregnancy for the first time. Among those mothers who have had antenatal care follow-up, nearly half (48 % of mothers for asphyxiated and 49.7 % of mothers for non-asphyxiated) had attended at hospital. Of all the respondents, five mothers (4.5 %) of cases and seventeen (7.8 %) mothers of the controls had one or more chronic medical illnesses (Table 2).

**Table 2:** Antepartum characteristics of the respondents among newborns in south Gondar zone public hospitals, Northwest Ethiopia 2021 (n = 329).

Variables	Case(%) (n = 110)	Controls(%) (n = 219)	Total(%)
Gravidity			
Primigravida	35(31.8)	81(36.9)	116(35.2)
Multigravida	75(68.2)	138(63.1)	213(64.8)
Parity			
Primipara	33(30.8)	75(35.2)	108(33.7)
Multipara	74(69.2)	138(64.8)	212(66.3)
Number of ANC			
0	12(10.9)	22(10)	34(10.3)
1-3	44(40)	83(37.9)	127(38.6)
4 and more	54(49.1)	114(52.1)	168(51.1)
Place of ANC			
Public hospital	47(48)	98(49.7)	145(49.2)
Private clinic	6(6.1)	18(9.1)	24(8.1)
Health center	45(45.9)	81(41.1)	126(42.7)
Danger sign/symptoms			
Yes	10(10.2)	32(16.2)	42(14.2)
No	88(89.8)	162(83.8)	252(85.8)
Chronic medical illness			
Yes	5(4.5)	17(7.8)	22(6.7)
No	105 (95.5)	202(92.2)	307(93.3)
Adverse birth outcome			
Yes	19(17.3)	52(23.7)	71(21.6)
No	91(82.7)	167(76.3)	258(78.4)

ANC: antenatal care.

### 3.3 Intra-partum related characteristics of respondents

Among all the respondents, majority (83.6 %) of cases and 77.2 % of controls, labor was started spontaneously. Instruments were applied to conduct the delivery of one-fifth (20 %) cases and twenty (9.1 %) controls. Nearly one-fourth (24.5 %) of cases and only 11 % of controls had experienced labor abnormalities and 81.5 % of labor abnormality among cases was at the second stage of labor (Table 3).

**Table 3:** Distribution of intrapartum related characteristics of the respondents among newborns in south Gondar zone public hospitals, Northwest Ethiopia 2021 (n = 329).

Variables	Cases (%) (n = 110)	Controls(%) (n = 219)	Total(%)
Type of labor			
Spontaneous	92(83.6)	169(77.2)	261(79.3)
Induced	18(16.4)	44(22.8)	68(20.7)
Fetal presentation			
Cephalic	88(80)	192(87.7)	280(85.1)
Non cephalic	22(20)	27(12.3)	49(14.9)
Mode of delivery			
Spontaneous vaginal	52(47.3)	145(66.2)	197(59.9)
Assisted breach	12(10.9)	19(8.7)	31(9.4)
Instrumental	22(20.0)	20(9.1)	42(12.8)
Cesarean section	24(21.8)	35(16.0)	59(17.9)
Birth attendant			
Intern	3(2.7)	10(4.6)	13(4)
Midwife	53(48.2)	127(58.0)	180(54.7)
GP	9(8.2)	16(7.3)	25(7.6)
IESO	33(30)	44(20.1)	77(23.4)
Obstetrician	12(10.9)	22(10)	34(10.3)
Labor abnormality			
Yes	27(24.5)	24(11)	51(15.5)
No	83(75.5)	195(89)	278(84.5)
Obstructed labor			
Yes	10(9.1)	5(2.3)	15(4.6)
No	100(90.9)	214(97.7)	314(95.4)
Prolonged ROM			
Yes	20(18.2)	49(22.4)	69(21.0)
No	90(81.8)	170(77.6)	260(79.0)
PROM			
Yes	9(8.2)	17(7.8)	26(7.9)
No	101(91.8)	202(92.2)	303(92.1)
Amniotic fluid status			
Meconium stained	33(30)	38(17.4)	71(21.6)
Clear	77(70)	181(82.6)	258(78.4)
Maternal pyrexia			
Yes	4(3.6)	4(1.8)	8(2.4)
No	106(96.4)	215(98.2)	321(97.6)
Cord prolapse			
Yes	12(10.9)	8(3.7)	20(6.1)
No	98(89.1)	211(96.3)	309(93.9)
Preeclampsia/Eclampsia			
Yes	28(25.5)	48(21.9)	76(23.1)
No	82(74.5)	171(78.1)	253(76.9)
APH			
Yes	31(28.2)	15(6.8)	46(14.0)
No	79(71.8)	204(93.2)	283(86.0)
Cause of APH			
Placental abruption	18(60)	13(81.25)	31(67.4)
Placenta previa	6(20)	2(12.5)	8(17.4)
Uterine rupture	6(20)	1(6.25)	7(15.2)
Safe childbirth checklist			
Yes	17(15.5)	30(13.7)	47(14.3)
No	93(84.5)	189(86.3)	282(85.7)
Nutritional status			
Undernourished	12(10.9)	14(6.4)	26(7.9)
Well nourished	98(89.1)	205(93.6	303(92.1)

APH: antepartum hemorrhage, GP: general practitioner, IESO: PROM, premature rupture of membrane.

### 3.4 Neonatal characteristics of the respondents

Among the study subjects, more than half (53.6 %) of cases and (50.2 %) of controls were male neonates. Among all study participants, 95.5 % of the cases and 97.3 % of controls were born singleton. One every four of the neonates (25.5 %) among cases and 19(8.7 %) of the controls were born with low birth weight. About 27.3 % of cases were born before 37 completed weeks of gestation. Whereas, only 9.1 % of controls were born before 37 completed weeks of gestation.

### 3.5 Factors associated with birth asphyxia

This study revealed that the determinant factors of birth asphyxia that have a p value < 0.05 were instrumental delivery, labor abnormality, cord prolapse, ante partum hemorrhage and preterm birth (Table 4).

**Table 4:** Bi-Variable and Multivariate Analysis for the determinants of birth Asphyxia among Newborns Delivered at Public hospitals of south Gondar zone, Northwest Ethiopia, 2021 (n = 329).

Variables	Cases (%)	Controls (%)	COR (95%CI)	AOR(95%CI)
Education level				
Unable to read and write	35(31.8)	52(23.7)	1.30(0.64,2.66)	1.39(0.51,3.78)
Able to read and write	17(15.5)	53(24.2)	0.62(0.28,1.37)	0.54(0.19,1.48)
Primary school	23(20.9)	51(23.3)	0.87(0.41,1.86)	0.94(0.36,2.43)
Secondary school	17(15.5)	28(12.8)	1.18(0.51,2.70)	0.94(0.34,2.61)
College and above	18(16.4)	35(16.0)	1	1
Danger sign of pregnancy				
Yes	10(10.2)	32(16.2)	0.58(0.27,1.25)	0.69(0.27,1.79)
No	88(89.8)	162(83.8)	1	1
Adverse birth outcome				
Yes	19(17.3)	52(23.7)	0.67(0.3,1.20)	0.76(0.35,1.65)
No	91(82.7)	167(76.3)	1	1
Type of labor				
Spontaneous	92(83.6)	169(77.2)	1	1
Induced	18(16.4)	44(22.8)	1.51(0.83,2.74)	0.48(0.21,1.08)
Fetal presentation				
Cephalic	88(80)	192(87.7)	1	1
Non cephalic	22(20)	27(12.3)	0.56(0.30,1.04)	1.38(0.49,3.89)
Mode of delivery				
Spontaneous vaginal delivery	52(47.3)	145(66.2)	1	1
Assisted breach	12(10.9)	19(8.7)	1.76(0.80,3.87)	2.08(0.64,6.74)
Instrumental	22(20.0)	20(9.1)	3.06(1.54,6.07)	3.19(1.23,8.36)*
Cesarean section	24(21.8)	35(16.0)	1.60(0.72,3.56)	1.14(0.42,3.11)
Birth attendant				
Intern	3(2.7)	10(4.6)	0.55(0.12,2.39)	3.76(0.55,25.45)
Midwife	53(48.2)	127(58.0)	0.76(0.35,1.65)	2.16(0.62–7.51)
GP	9(8.2)	16(7.3)	1.03(0.35,3.03)	0.78(0.15–4.07)
IESO	33(30)	44(20.1)	1.37(0.59,3.17)	2.15(0.64,7.27)
Obstetrician	12(10.9)	22(10)	1	1
Labor abnormality				
Yes	27(24.5)	24(11)	2.64(1.44,4.85)	3.24(1.31,8.03)*
No	83(75.5)	195(89)	1	1
Obstructed labor				
Yes	10(9.1)	5(2.3)	4.2(1.42,12.85)	1.22(0.13,11.88)
No	100(90.9)	214(97.7)	1	1
Amniotic fluid status				
Meconium stained	33(30)	38(17.4)	2.04(1.19,3.49)	2.02(0.96,4.25)
Clear	77(70)	181(82.6)	1	1
Cord prolapse				
Yes	12(10.9)	8(3.7)	3.23(1.28,8.15)	7.06(2.25,22.12)**
No	98(89.1)	211(96.3)	1	1
APH				
Yes	31(28.2)	15(6.8)	5.34(2.73,10.41)	4.68(2.00,10.95)***
No	79(71.8)	204(93.2)	1	1
Nutritional status				
Undernourished	12(10.9)	14(6.4)	1.79(0.79,4.02)	1.56(0.52,4.63)
Well nourished	98(89.1)	205(93.6	1	1
Birth weight				
Low birth weight	28(25.5)	19(8.7)	3.59(1.90,6.79)	2.68(0.79,9.08)
Normal birth weight	82(74.5)	200(91.4)	1	1
GA				
<37 weeks	30(27.3)	20(9.1)	3.48(1.96,6.27)	3.84(1.32,11.20)*
≥37 weeks	80(72.7)	199(90.9)	1	1

APH- antepartum hemorrhage; GA-gestational age; ETB- Ethiopian birr; COR-crude odds ratio; AOR-adjusted odds ratio; *p value of< 0.05; **p value < 0.001; ***p value < 0.0001.

The likelihood of developing birth asphyxia among neonates by instrumental delivery was 3.19 times more than those delivered with spontaneous vaginal delivery (AOR: 3.19, CI: 1.23, 8.36). The odds of having birth asphyxia was 3.24 times more likely among neonates born from mothers who had labor abnormalities than those who had no labor abnormalities (AOR: 3.24, CI: 1.31, 8.03)**.** The odds of having birth asphyxia was 7.06 times higher among neonates born from mothers who had cord prolapse than their counterparts (AOR: 7.06, CI: 2.25, 22.12)**.** The likelihood of having birth asphyxia among newborns from mothers who had ante partum hemorrhage was 4.68 times higher than their counter parts(AOR: 4.68, CI: 2.00, 1095). The odds of developing birth asphyxia were 3.84 times higher among preterm neonates than their counterparts (AOR: 3.84, CI: 1.32, 11.20)**.**

## 4 Discussion

This study revealed that instrumental delivery, the presence of labor abnormalities, cord prolapse, ante partum hemorrhage and preterm birth were found to be associated with birth asphyxia.

The likelihood of developing birth asphyxia among neonates with instrumental delivery was 3.19 times more than those delivered with spontaneous vaginal delivery. A previous study at Amhara region referral hospitals noted that the odds of developing birth asphyxia of newborns with instrumental delivery were 3.03 times more likely than those delivered by spontaneous vaginal delivery [13]. Our study finding is also consistent with the studies conducted at Addis Ababa [20], Dire Dawa [21], Ethiopia [22], Indonesia [23], Australia [24], where the odds of acquiring birth asphyxia were between one and five. This could be due to the fact that one of the indications of applying instruments (forceps or vacuum) is when there is evidence of a non-reassuring fetal condition exists. So, when there is an insulted fetal condition at the second stage of labor they are highly prone to instrumental delivery. On the other hand, applying instruments can cause birth trauma, which further leads to birth asphyxia [25].

The odds of having birth asphyxia were 3.24 times more likely among neonates born from mothers who had labor abnormality than those who had no labor abnormality. This finding is harmonious with those studies done previously at Amhara region [13], Gondar [14], Dire Dawa [21], Hosaena [26], Ethiopia [27], Colombia [28], Nova-Scotia [29], where the odds of developing birth asphyxia found to be between two and thirty two. The possible explanation is when the labor progress goes abnormally; the mother becomes prone to dehydration, cephalopelvic disproportion and uterine rupture and maternal obstetric trauma in general. This causes utero-placental insufficiency to happen and ultimately results in reduced fetal oxygenation.

The findings of this study showed that the odds of having birth asphyxia were 7.06 times higher among neonates born from mothers who had cord prolapse than their counterparts. This study is in line with a study done at Karachi hospital, Pakistan [18], United States of America [30]. A retrospective cohort study conducted in United States of America also showed the same finding. Umbilical cord prolapse during labor and delivery is associated with perinatal morbidity and neonatal mortality [31]. This is explained by the prolapsed cord is vulnerable to compression, umbilical vein occlusion, and umbilical artery vasospasm, which can compromise the fetal oxygenation.

According to our study, the likelihood of having birth asphyxia among newborns from mothers who had ante partum hemorrhage were 4.68 times higher than their counter parts. This study finding is consistent with the previous studies done at central Tigray [32], Addis Ababa [20], Ethiopia [23], Universitario del Valle, Cali, Colombia [22]. The association between APH and birth asphyxia can be explained by the fact that maternal hemorrhage causes utero-placental insufficiency which leads to reduced blood flow from the placenta to the fetus. This ultimately results in fetal hypoxemia and birth asphyxia. On the other hand, this might be explained as mothers with ante partum hemorrhage are prone to therapeutic termination of the pregnancy before the age of fetal viability, which further aggravates the difficulty in cardiopulmonary transition of the neonate [33].

Furthermore, preterm birth is found to be independently associated with birth asphyxia. The odds of developing birth asphyxia were 3.84 times higher among preterm neonates than their counterparts. This finding is in line with previous studies done at Addis Ababa [20], central Tigray [32], Hosaena [27], Ethiopia [23]. This is due to the fact that preterm neonates have insufficient surfactant production, which leads to inadequate pulmonary gas exchange and ultimately results in difficulty of breathing from impaired cardiopulmonary transition.

## 5 Conclusion

Until today, birth asphyxia is a global health problem of neonates and has become the leading cause of neonatal morbidity and mortality because of the preventable antepartum and intra-partum complications of pregnancy. In this study: Antepartum hemorrhage, labor abnormalities, cord prolapse, instrumental delivery and preterm birth were found to be independently associated with the development of birth asphyxia. Healthcare providers in obstetric care should prioritize preventive measures for both antenatal and intra-partum obstetric complications. Additionally, it is crucial to administer early interventions with precision to address potential complications before they arise.

### 5.1 Strength and limitation

A systematic sampling method was used and it is possible to generalize the findings to the source population. Unlike other studies conducted before, our study has incorporated neonates with birth asphyxia at the labor ward whom do not need a referral to NICU after simple resuscitation has been given. In addition to this, the associations of new variables such as WHO safe child birth checklist and labor abnormality were studied. The case control nature of the study design used and its retrospective nature in this study make it vulnerable for recall bias. Despite appropriate randomization to select the study participants and making the questions neutral and appropriate options has been made, the study is vulnerable to selection and response bias.

## Ethical statement and consent to participate

The study protocol has been examined and approved by University Gondar Institutional Review Board (IRB) with reference number: SMIDW/18/2013 EC. Prior to data collection, written informed consent was obtained from study participants and actual data collection was conducted after the hospital has permitted to do so. This study was done in line with the Helsinki's declaration. Confidentiality is kept throughout the study.

## Data availability statement

Almost all of the data set is used in the manuscript; however, if someone wants to get the raw data, it is possible to get it from the corresponding author ESL: *eyobshitie@gmail.com* upon rational request.

## CRediT authorship contribution statement

**Eyob Shitie Lake:** Writing – review & editing, Writing – original draft, Software, Methodology, Formal analysis, Data curation, Conceptualization. **Zinie Abita:** Writing – review & editing. **Besfat Berihun Erega:** Writing – review & editing.

## Declaration of competing interest

The authors declare that they have no known competing financial interests or personal relationships that could have appeared to influence the work reported in this paper.
